# Sarcopenia risk and associated factors among Chinese community-dwelling older adults living alone

**DOI:** 10.1038/s41598-021-01614-7

**Published:** 2021-11-15

**Authors:** Li Cheng, Janet W. H. Sit, Helen Y. L. Chan, Kai Chow Choi, Regina K. Y. Cheung, Martin M. H. Wong, Francis Y. K. Li, Tin Yan Lee, Elina S. M. Fung, Keen Man Tai, Winnie K. W. So

**Affiliations:** 1 The School of Nursing, Sun Yat-Sen University, Guangzhou, China; 2 The Nethersole School of Nursing, Faculty of Medicine, The Chinese University of Hong Kong, Shatin, N.T., Hong Kong, Special Administrative Region China; 3 The Neighbourhood Advice-Action Council, North Point, Hong Kong, Special Administrative Region China

**Keywords:** Health care, Risk factors

## Abstract

Sarcopenia, defined as a progressive loss of muscle mass and reduced muscle strength and functional capacity, is common among older adults. This study aimed to assess the proportion of people at risk of sarcopenia and probable sarcopenia among Chinese community-dwelling older adults living alone and to identify the associated factors. A total of 390 older adults were included in this study. Sarcopenia and probable sarcopenia were defined according to the criteria of the Asian Working Group for Sarcopenia 2019. Data on socio-demographic characteristics, health status, health behaviours and lifestyle characteristics, nutritional status, physical activity level, and depressive symptoms were collected. The association between these characteristics and sarcopenia risk was analysed using a multivariate ordinal logistic regression. The proportion of subjects at risk of sarcopenia and probable sarcopenia was found to be 57.7% and 30%, respectively. Older age, being malnourished and being at risk of malnutrition were significantly associated with sarcopenia risk. Being educated to secondary level or above, being overweight or obese and higher physical activity level were associated with decreased sarcopenia risk. Our results showed that older adults living alone were at high risk of developing sarcopenia and probable sarcopenia. These results emphasise the urgent need to initiate aggressive screening and holistic lifestyle therapeutic intervention strategies for this high-risk population.

## Introduction

Sarcopenia is characterised by age-related decreased skeletal muscle mass, diminished muscle strength, and deteriorated physical performance^1^. It is considered a reliable predictor of poorer prognoses among older adults, and is associated with functional impairment, increased risk of cardiometabolic diseases and falls and fractures, limited mobility, and poor quality of life^1,2^. Sarcopenia also increases the risk of institutionalisation and hospitalisation and significantly increases healthcare expenditure. Sarcopenia, however, is often underdetected and undertreated in mainstream practice. Consensus updates of both the European Working Group on Sarcopenia in Older People (EWGSOP2) and the Asian Working Group for Sarcopenia (AWGS) 2019 highlighted the importance of developing methods for the early identification of those at risk for sarcopenia in the community setting without advanced diagnostic tests^1,3^. Consensus updates also introduced the term ‘probable sarcopenia’, defined as low muscle strength with or without reduced physical performance. In view of the reversible nature of this condition, early detection of older adults with probable sarcopenia could facilitate timely education, implementation of lifestyle interventions, and referral for confirmatory diagnosis.

The increasing number of older adults living alone is a growing global challenge. Owing to the recent improvements in the housing quality, living alone may have become a preferred alternative living arrangement for older adults, but it may often be an inevitable arrangement when they become single, divorced, or widowed, or having no informal caregivers looking after them. Living alone is usually accompanied with feelings of loneliness and insecurity and difficulties in daily living, all of which pose enormous challenges for older adults^4^. Evidence consistently supports that living alone is associated with increased risks of malnutrition, a declining health status, depressive symptoms, hospital readmission, and a deteriorated quality of life^5,6^. Despite these drawbacks, in 2012, nearly 29% of the 46 million community-dwelling older adults in the United States lived in single-person households, and more than half of the community-dwelling old persons (≥ 80 years old) were living alone^4^. In Europe, the proportion of older adults living alone has increased by 16% over the past two decades, with more than one-third of people aged > 65 years currently living alone^7^. It was estimated that Chinese older population aged 65 years and above had reached 158 million in 2017 and 13.1% of them were reported to be living alone^8^. This number is expected to increase due to population ageing, socioeconomic development, and family nuclearisation. Older adults living alone tend to have a higher risk of developing sarcopenia than those who are not, as indicated by the results of univariate analyses in previous studies^9^. The sarcopenia status of older adults living alone warrants immediate attention and investigation.

The risk of sarcopenia may be more salient for Chinese older adults living alone than for those in Western communities. Because Chinese collectivist culture values family-centeredness and social cohesion, Chinese older adults can often experience stigma, discrimination, and depressive symptoms when living alone^6,10^. To date, several studies have reported on the prevalence of sarcopenia in the Chinese community. Liu et al. reported a prevalence of 19.3% among 4500 multi-ethnic adults aged ≥ 50 years, according to the AWGS 2014 criteria^9^. In another study, approximately 3.8% of 3997 community-dwelling older adults in Hong Kong were determined to have sarcopenia using the SARC-F questionnaire^11^. However, the population of Chinese older adults who live alone remains underrepresented, and there is a paucity of studies addressing the prevalence of sarcopenia and probable sarcopenia using the updated consensus criteria.

The pathophysiology of sarcopenia is multifaceted, and several factors have been investigated with respect to their role in the development of sarcopenia^9,12,13^. However, the reported associations of socio-demographic factors, health behaviours, and psychological factors with the risk of sarcopenia are inconsistent across studies due to heterogeneity in the study design, the operational definition of sarcopenia, the target population, and the factors analysed. Moreover, few studies have taken probable sarcopenia into account when exploring the potential determinants of sarcopenia. Identifying the associated factors, especially modifiable factors, for those at risk of sarcopenia is imperative to inform the development of therapeutic interventions tailored to mitigate sarcopenia among older adults living alone.

Therefore, the aims of the present secondary analysis were (1) to report the proportion of Chinese older adults living alone who were at risk of ‘sarcopenia’ and ‘probable sarcopenia’ using the SARC-F, CC, and SARC-CalF, according to the updated definition proposed by the AWGS 2019, and (2) to identify factors associated with an increased odds of sarcopenia.

## Methods

### Study design and population

This study was a secondary data analysis of a population-based cross-sectional study, with the aim to investigate the nutritional status, health behaviours, lifestyle characteristics, and health status of the community-dwelling older population in Hong Kong. This study was conducted from May to September 2017. Details of the location, study population, and data collection methodology have been published elsewhere^10^. Briefly, the inclusion criteria were an age > 60 years, living at home, and an ability to communicate in Chinese and provide written informed consent. Eligible participants were contacted via phone and recruited through a large registered charitable NGO. Trained interviewers conducted face-to-face interviews using a standardised questionnaire. In total, 1179 eligible older adults living alone were successfully contacted by phone, and 613 of them provided informed consent to participate in this study (response rate = 52.0%).

### Measurement

#### Assessment of sarcopenia

According to AWGS2019 guideline^3^, participants at risk of sarcopenia were identified using the SARC-F (≥ 4), CC (< 34 cm in men, < 33 cm in women), or SARC-CalF (≥ 11).

The SARC-F examines five domains, namely strength, assistance in walking, rising from a chair, climbing chairs, and falling episodes, and participants were asked how much difficulty they faced when performing those activities in the past year. Each item was scored from 0 (no difficulties) to 2 (a lot of difficulties) based on the participant’s response. The total score was calculated by summing the participant’s responses to each item, and participants with a SARC-F score ≥ 4 were considered to have a high risk of sarcopenia. The SARC-F has been validated in a Hong Kong Chinese population and has excellent specificity and negative predictive value but a low sensitivity^11^.

The SARC-CalF scale is composed of six items: the first five items are identical to and scored the same as those in the SARC-F, and the sixth item is the CC (measurement of the right calf in a standing position at the point of greatest circumference). The CC was measured using an anthropometric measuring tape. The cut-off points for the CC are 34 cm for men and 33 cm for women^3^. The CC in the SARC-CalF is scored as 0 points if it is above the cut-off point and as 10 points it is below or equal to the cut-off point (suggesting low muscle mass). The SARC-CalF score ranges from 0 to 20 points, and a total score of ≥ 11 is considered to indicate a risk of sarcopenia^14^.

#### Assessment of probable sarcopenia

Probable sarcopenia was characterised by a decline in muscle strength. Handgrip strength was measured by a trained research nurse using a handheld dynamometer (TTM Smedley's Dynamometer, Japan). Each participant stood upright with the arm vertical and the dynamometer close to the body. The grip of each hand was measured, and the average value of the two handgrip measurements was presented as the maximal grip strength^15,16^. Weak handgrip strength (indicating probable sarcopenia) was defined as < 28 kg in men and < 18 kg in women^3^.

### Socio-demographic characteristics

Socio-demographic characteristics, including age, sex, marital status, educational level, employment status, monthly household income, and receipt of comprehensive social security assistance, were recorded.

### Health status

Health status was determined by the Charlson comorbidity index (CCI) score and the presence of other common geriatric conditions. The participants were asked if they had ever been diagnosed with one or more of the following illnesses: myocardial infarction, angina, heart failure, peripheral vascular disease, cerebrovascular diseases, high blood pressure, dyslipidaemia, chronic lung diseases, diabetes, liver diseases, cancers, arthritis, and Parkinson’s disease. The CCI represents the burden of comorbidities, and the total CCI score was calculated by summing the weighted scores of each reported condition of the individual^17^. Other common geriatric conditions, such as visual and hearing abilities (subjective clear, fair, and unclear) and appetite (good, normal, and poor), were rated by the participants on a 3-point Likert scale. The use of dentures and chewing difficulty were dichotomised (yes/no). The BMI was calculated as the weight in kilograms divided by the height in meters squared and categorised as underweight (≤ 18.5 kg/m^2^), normal weight (18.5–23.9 kg/m^2^), overweight (24.0–27.9 kg/m^2^), and obese (≥ 28.0 kg/m^2^)^18^.

### Health behaviours and lifestyle characteristics

Health behaviours and lifestyle characteristics included the level of physical activity, dietary intake, and smoking and drinking habits.

The International Physical Activity Questionnaire Short Form (IPAQ-SF) is one of the most widely used self-report questionnaires to assess the level of physical activity. It consists of seven items and captures the daily time spent in sitting, walking, and engaging in moderate and vigorous physical activity during the last 7 days. The metabolic-equivalent minutes are calculated by multiplying the amount of time spent in the activity by 8 (for vigorous-intensity activities), 4 (for moderate-intensity activities), or 3.3 (for walking)^19^. The participants were categorised into low-, moderate-, and high-activity categories according to their energy expenditure per week. The Chinese IPAQ-SF has been validated against physical activity logs and has satisfactory reliability^20^.

The consumption of five major food groups (grains, vegetables, fruit, meat, and milk) was assessed using a culturally relevant food frequency questionnaire^21^. A booklet with detailed instructions, together with standard-size models of bowls, cups, and foods, were provided to illustrate the serving sizes during the interviews. Compliance with the dietary guidelines was determined by checking the dietary consumption against the recommendations of the Healthy Eating Food Pyramid for Elderly in Hong Kong.

Information on smoking (never, former smoker, or current smoker) and alcohol consumption (never, former drinker, or current drinker) statuses was directly reported by the participants.

### Nutritional status

The global nutritional status of the participants was measured using the 18-item MNA. This tool assesses four aspects: (1) anthropometric conditions, including BMI, mid-arm circumference, CC, and weight loss; (2) general conditions, including lifestyle, medication, stress morbidity, and presence of neuro-psychological problems; (3) dietary conditions, including number of meals, autonomy in feeding, food and fluid intake, and mode of feeding; and (4) self-perceived health-status conditions, including self-perception of health and nutritional statuses. Individuals were classified into three groups using threshold values of < 17 for ‘malnutrition’, 17–23.5 for ‘at risk of malnutrition’, and 24–30 for ‘normal nutritional status’. The MNA has been validated to screen Chinese geriatric patients for malnutrition and has satisfactory internal consistency (Cronbach’s α = 0.798), and this instrument was suggested in previous reviews to be a useful tool for screening malnourished older adults and those at risk of malnutrition^22^.

### Depressive symptoms

The 4-item version of the Chinese Geriatric Depression Scale (GDS-4) was used to measure depressive symptoms, with scores ranging from 0 to 4. Participants with a score of ≥ 2 were considered to have depressive symptoms. The GDS-4 has a better diagnostic ability, with a sensitivity of 61% and a specificity of 81%, than a comprehensive diagnostic interview^23^.

### Data collection

Eligible participants were approached and provided with information sheets. After obtaining their written informed consent, face-to-face structured interviews were conducted by well-trained interviewers to collect participants’ data. To ensure accurate assessment, all interviewers had received standardised protocol-oriented training in conducting structured interviews and anthropometric measurements before the commencement of the study.

### Statistical analysis

Data analysis was performed using IBM SPSS Statistics for Windows, Version 24.0. Continuous variables are presented as the means (standard deviations) for normally distributed data. As a general of thumb, data were considered as normal if the absolute values of skewness and kurtosis are less than 2^24^. Categorical variables are reported as frequencies (percentages). The participants were classified as having sarcopenia risk, probable sarcopenia, or a low risk of sarcopenia according to the AWGS 2019 criteria. Socio-demographic characteristics, health status, health behaviours, lifestyle characteristics, and nutritional status were presented and compared using a one-way analysis of variance, Pearson’s chi-square test, or Fisher’s exact test, as appropriate. Factors with a *p*-value < 0.25 in univariate analyses were selected for a subsequent multivariate ordinal logistic regression analysis^25^. This less stringent *p*-value was selected to enable a broader inclusion of potential variables to determine the contribution of each variables to sarcopenia risk, thereby yielding more accurate results in identifying the associated factors. The odds ratio, 95% confidential interval, and *p* value were reported to determine the contribution of factors to the risk of sarcopenia. Two-tailed statistical tests were conducted, and the level of significance was set at 0.05.

### Ethics approval and consent to participate

This study was conducted according to the Declaration of Helsinki and approved by the Survey and Behavioural Research Ethics Committee of the Chinese University of Hong Kong. Confidentiality of subjects’ personal details was ensured by having the NGO staff contacting the eligible subjects during subject recruitment. Subjects were informed about the study details, the anonymity of any data collected, and their rights to withdraw from the study at any time through an information sheet. Subjects expressing an interest in study participation were enrolled only if they had provided written informed consent. We also obtained prior approval for using the instruments for data collection.

## Results

### Participant characteristics

Among the 613 participants who met the inclusion criteria for this secondary analysis, 390 participants who lived alone completed the SARC-F survey and had their CC measured. No missing data were reported. The proportion of subjects at risk of sarcopenia and those having probable sarcopenia, defined according to the AWGS 2019 criteria, was 57.7% and 30%, respectively. The mean age of the participants was 78.1 ± 7.4 years. The majority of the participants were female (58.0%), single/divorced/separated/widowed (77.7%), and retired (95.4%) (Table 1). The majority of the participants had only primary or lower educational attainment (73.1%) and 20% reported CCI score above 2. BMI assessment showed about half (57.4%) of participants were either overweight or obese, and 7.4% were underweight. Only 12.3% of the participants reported a low level of physical activity, and nearly one-third had a Mini Nutritional Assessment (MNA) score less than 24, indicating that they were at risk of malnutrition (27.4%) or already malnourished (0.8%).

**Table 1:** Characteristics of the participants who live alone (N = 390).

	All (N = 390)	At risk of sarcopenia (n = 225)	Probable sarcopenia (n = 117)	Low risk of sarcopenia (n = 48)	*p*-value*
**Socio-demographic characteristics**					
Age (years) [range: 61–106], mean (SD)	78.1 (7.4)	79.5 (7.7)	77.0 (6.7)	74.7 (6.5)	< 0.001
Sex^a^, n (%)					0.921
Male	164 (42.0)	93 (41.3)	51 (43.6)	20 (41.7)	
Female	226 (58.0)	132 (58.7)	66 (56.4)	28 (58.3)	
Marital status, n (%)					0.787
Single/divorced/separated/ widowed	303 (77.7)	172 (76.4)	93 (79.5)	38 (79.2)	
Married/cohabited	87 (22.3)	53 (23.6)	24 (20.5)	10 (20.8)	
Educational level, n (%)					0.021
No formal education	127 (32.6)	87 (38.7)	28 (23.9)	12 (25.0)	
Primary school	158 (40.5)	88 (39.1)	52 (44.5)	18 (37.5)	
Secondary school or above	105 (26.9)	50 (22.2)	37 (31.6)	18 (37.5)	
Employment status, n (%)					0.443
Retired	372 (95.4)	211 (94.6)	113 (96.6)	46 (95.8)	
Unemployed	12 (3.1)	8 (3.6)	2 (1.7)	2 (4.2)	
Have part-time/full-time job	6 (1.6)	4 (1.8)	2 (1.7)	0	
Received CSSA^b^, n (%)					0.315
No	144 (36.9)	87 (38.7)	44 (37.6)	13 (27.1)	
Yes	246 (63.1)	138 (61.3)	73 (62.4)	35 (72.9)	
**Health status**					
Charlson comorbidity index (CCI), mean (SD)	1.0 (1.8)	1.1 (2.2)	0.9 (1.4)	0.9 (1.4)	0.104
History of hypertension, n (%)					0.604
No	144 (36.9)	87 (38.7)	42 (35.9)	15 (31.2)	
Yes	246 (63.1)	138 (61.3)	75 (64.1)	33 (68.8)	
History of diabetes, n (%)					0.249
No	278 (71.3)	164 (72.9)	77 (65.8)	37 (77.1)	
Yes	112 (28.7)	61 (27.1)	40 (34.2)	11 (22.9)	
Visual impairment, n (%)					0.003
No	164 (42.1)	87 (38.6)	50 (42.7)	27 (56.3)	
A little	129 (33.1)	69 (30.7)	41 (35.0)	19 (39.6)	
A lot	97 (24.8)	69 (30.7)	26 (22.2)	2 (4.2)	
Hearing impairment, n (%)					0.001
Clear	238 (61.0)	130 (57.8)	67 (57.3)	41 (85.4)	
Fair	99 (25.4)	56 (24.9)	38 (32.5)	5 (10.4)	
Unclear	53 (13.6)	39 (17.3)	12 (10.2)	2 (4.2)	
Use of denture					0.849
No	135 (34.7)	78 (34.8)	42 (35.9)	15 (31.3)	
Yes	254 (65.3)	146 (65.2)	75 (64.1)	33 (68.8)	
Difficulty in chewing food, n (%)					0.093
No	239 (61.3)	129 (57.3)	75 (64.1)	35 (72.9)	
Yes	151 (38.7)	96 (42.9)	42 (35.9)	13 (27.1)	
Appetite, n (%)					0.046
Good	219 (56.2)	117 (52.0)	68 (58.1)	34 (70.8)	
Normal	153 (39.2)	98 (43.6)	43 (36.8)	12 (25.0)	
Bad	18 (4.6)	10 (4.4)	6 (5.1)	2 (4.2)	
BMI	24.0 (3.9)	22.9 (3.7)	25.4 (3.9)	25.6 (3.3)	< 0.001
BMI (kg/m^2^)					< 0.001
Underweight (≤ 18.5)	29 (7.4)	27 (12.0)	2 (1.7)	0	
Normal (18.5–23.9)	137 (35.1)	98 (43.6)	27 (23.1)	12 (25.0)	
Overweight (24.0–27.9)	75 (19.2)	36 (16.0)	30 (25.6)	9 (18.8)	
Obesity (≥ 28.0)	149 (38.2)	64 (28.4)	58 (49.6)	27 (56.3)	
**Health behaviors and lifestyle characteristics**					
Smoking status, n (%)					0.296
Non-smoker	286 (73.3)	168 (74.7)	88 (75.2)	30 (62.5)	
Ex-smoker	60 (15.4)	30 (13.3)	18 (15.4)	12 (25.0)	
Current smoker	44 (11.3)	27 (12.0)	11 (9.4)	6 (12.5)	
Drinking status, n (%)					0.474
Non-drinker	306 (78.5)	177 (78.7)	94 (80.3)	35 (72.9)	
Ex-drinker	46 (11.8)	28 (12.4)	11 (9.4)	7 (14.6)	
Current drinker	38 (9.7)	20 (8.9)	12 (10.3)	6 (12.5)	
Level of physical activity (IPAQ-SF)					0.144
Low	48 (12.3)	31 (13.8)	14 (12.0)	3 (6.3)	
Moderate	227 (60.5)	143 (63.5)	66 (56.4)	27 (56.3)	
High	106 (27.2)	51 (22.7)	37 (31.6)	18 (37.5)	
Dietary intake: below recommendation of the dietary guidelines					
Grains (3 to 5 bowls per day)	139 (35.6)	84 (37.3)	41 (35.0)	14 (29.2)	0.531
Vegetables (at least 3 servings per day)	330 (84.6)	190 (84.4)	103 (88.0)	37 (77.1)	0.207
Fruits (at least 2 servings per day)	286 (73.3)	167 (74.2)	87 (74.4)	32 (66.7)	0.537
Meats (5 to 6 taels per day)	369 (94.6)	215 (96.0)	108 (92.3)	46 (95.8)	0.516
Milk (1 to 2 glass per day)	316 (81.0)	179 (79.9)	99 (84.6)	38 (79.2)	0.479
**Nutritional status**					
Mini Nutritional Assessment (MNA) score, mean (SD)	25.0 (2.7)	24.1 (2.9)	25.1 (2.4)	26.3 (2.0)	< 0.001
Malnutrition status, n (%)					< 0.001
Normal	280 (71.8)	138 (61.3)	100 (85.5)	42 (87.5)	
At risk	107 (27.4)	84 (37.3)	17 (14.5)	6 (12.5)	
Malnourished	3 (0.8)	3 (1.3)	0	0	
**Depressive symptoms**					0.485
No	320 (82.1)	179 (79.6)	99 (84.6)	41 (85.4)	
Yes	70 (17.9)	46 (20.4)	18 (15.4)	7 (14.6)	
**Sarcopenia-relevant parameters**					
SARC-F score	1.8 (1.9)	2.39 (2.2)	1.1 (1.1)	0.7 (0.9)	< 0.001
Calf circumference (cm)	33.1 (3.5)	31.2 (3.1)	35.5 (2.1)	35.9 (2.6)	< 0.001
Dominant handgrip strength (kg)	17.7 (7.8)	15.9 (7.6)	17.7 (6.6)	25.9 (6.5)	< 0.001
Non-dominant handgrip strength (kg)	16.4 (7.5)	14.7 (6.9)	15.9 (6.4)	25.4 (6.3)	< 0.001

^a^Sex distribution of the Hong Kong population aged 60 or above: males, 643,258 (47.6%); females, 707,438 (52.4%).

^b^CSSA: comprehensive social security assistance; is a governmental financial assistance scheme providing to support individuals to meet their basic needs.

*Continuous and categorical variables were compared among the three groups using one-way ANOVA and chi-square test respectively.

### Factors associated with risk of sarcopenia

The results of the univariate analysis are presented in Table 2. Older age, visual and hearing impairment, difficulty in chewing food, fair appetite, and risk of malnutrition were factors associated with an increased risk of sarcopenia. However, higher educational attainment and high physical activity level were associated with decreased sarcopenia risk. Participants with a higher body mass index (BMI) had a lower risk of sarcopenia than those with a normal weight [overweight, odds ratio (OR) 0.38; 95% confidence interval (CI) 0.21, 0.67; *p* = 0.001; obesity, OR 0.30; 95% CI 0.19, 0.49; *p* < 0.001). In contrast, underweight participants had a higher risk of sarcopenia than participants with a normal weight (OR 5.22; 95% CI 1.17, 23.41; *p* = 0.031).

**Table 2:** Factors associated with risk of sarcopenia estimated by univariate ordinal logistic regression analysis (N = 390).

	OR (95% CI)	*P* value
**Socio-demographic characteristics**		
Age (per 10 years)	1.89 (1.42, 2.50)	< 0.001
Sex		
Male (ref)	1	
Female	0.95 (0.64, 1.40)	0.788
Marital status		
Single/divorced/separated/ widowed (ref)	1	
Married/cohabited	0.85 (0.53, 1.37)	0.508
Educational level		
No formal education (ref)	1	
Primary school	0.61 (0.38, 0.98)	0.040
Secondary school or above	0.43 (0.25, 0.71)	0.001
Employment status		
Retired (ref)	1	
Unemployed	1.33 (0.41, 4.30)	0.633
Have part-time/full-time job	1.70 (0.30, 9.51)	0.549
Monthly household income (HK$)		
< 6000 (ref)	1	
≥ 6000	3.05 (0.83, 11.16)	0.092
Received CSSA^a^		
No (ref)	1	
Yes	1.26 (0.84, 1.89)	0.267
**Health status**		
Charlson comorbidity index (CCI)	1.15 (0.99, 1.33)	0.071
History of hypertension, n (%)		
No (ref)	1	
Yes	0.14 (0.02, 0.16)	0.068
History of diabetes, n (%)		
No (ref)	1	
Yes	1.10 (0.72, 1.69)	0.668
Visual impairment, n (%)		
No (ref)	1	
A little	1.05 (0.67, 1.63)	0.843
A lot	2.43 (1.43, 4.13)	0.001
Hearing impairment, n (%)		
Clear (ref)	1	
Fair	1.30 (0.82, 2.06)	0.264
Unclear	2.55 (1.32, 4.91)	0.005
Use of denture		
No (ref)	1	
Yes	1.04 (0.69, 1.57)	0.846
Difficulty in chewing food, n (%)		
No (ref)	1	
Yes	1.55 (1.03, 2.33)	0.036
Appetite, n (%)		
Good (ref)	1	
Fair	1.63 (1.08, 2.47)	0.020
Bad	1.15 (0.45, 2.94)	0.764
Body Mass Index (BMI, kg/m^2^)		
Normal (ref)	1	
Underweight (≤ 18.5)	5.22 (1.17, 23.41)	0.031
Overweight (24.0–27.9)	0.38 (0.21, 0.67)	0.001
Obese (≥ 28.0)	0.30 (0.19, 0.49)	< 0.001
**Health behaviors and lifestyle characteristics**		
Smoking status		
Non-smoker (ref)	1	
Ex-smoker	0.64 (0.38, 1.09)	0.097
Current smoker	1.04 (0.55, 1.96)	0.896
Drinking status		
Non-drinker (ref)	1	
Ex-drinker	1.04 (0.57, 1.93)	0.889
Current drinker	0.78 (0.41, 1.49)	0.463
Level of physical activity		
Low (ref)	1	
Moderate	0.79 (0.41, 1.49)	0.463
High	0.49 (0.24, 0.97)	0.040
Dietary intake: below recommendation of the dietary guidelines		
Grains (3 to 5 bowls per day)	0.82 (0.54, 1.23)	0.337
Vegetables (at least 3 servings per day)	0.91 (0.53, 1.55)	0.730
Fruits (at least 2 servings per day)	0.85 (0.55, 1.32)	0.479
Meats (5 to 6 taels per day)	0.30 (0.69, 1.63)	0.401
Milk (1 to 2 glass per day)	1.16 (0.70, 1.92)	0.570
**Nutritional status**		
Normal (ref)	1	
At risk or malnourished	3.82 (2.29, 6.35)	< 0.001
**Depressive symptoms**	0.73 (0.43, 1.23)	0.251

*OR* odds ratio, *CI* confidence interval, *ref* Reference group of the categorical variable.

^a^*CSSA* Comprehensive Social Security Assistance.

The results of a multivariate ordinal logistic regression analysis are provided in Table 3. Older age [adjusted OR (AOR) per 10-year increment: 1.68; 95% CI 1.17, 2.41; *p* = 0.005] and being at risk of malnutrition or malnourished (AOR 2.25; 95% CI 1.19, 4.29; *p* = 0.013) were significantly associated with an increased odds of having sarcopenia. Secondary school or higher educational attainment (AOR 0.50; 95% CI 0.26, 0.96; *p* = 0.038) and a high level of physical activity (AOR 0.36; 95% CI 0.16, 0.86; *p* = 0.020) were negatively associated with the risk of developing sarcopenia. Furthermore, after controlling for confounders in our multivariate analysis, being overweight and obese remained as a factor associated with a lower risk of sarcopenia (overweight: AOR 0.39; 95% CI, 0.20, 0.76; *p* = 0.006; obesity: AOR 0.27; 95% CI 0.15, 0.47; *p* < 0.001).

**Table 3:** Factors associated with risk of sarcopenia estimated by multivariable ordinal logistic regression analysis (N = 390).

	Odds ratio (95% CI)	*p*-value
**Socio-demographic characteristics**		
Age per 10 years	1.68 (1.17, 2.41)	0.005
Educational level		
No formal education (ref)	1	
Primary school	0.65 (0.36, 1.18)	0.160
Secondary school or above	0.50 (0.26, 0.96)	0.038
Monthly household income (HK$)		
< 6000 (ref)		
≥ 6000	1.07 (0.25, 4.65)	0.930
Charlson comorbidity index (CCI)	1.18 (0.99, 1.42)	0.070
History of hypertension, n (%)		
No (ref)	1	
Yes	0.69 (0.41, 1.15)	0.153
Visual impairment, n (%)		
No (ref)	1	
A little	0.71 (0.42, 1.21)	0.210
A lot	1.88 (0.94, 3.75)	0.072
Hearing impairment, n (%)		
Clear (ref)	1	
Fair	0.94 (0.52, 1.67)	0.820
Unclear	1.26 (0.53, 2.99)	0.604
Difficulty in chewing food, n (%)		
No (ref)	1	
Yes	1.48 (0.90, 2.42)	0.118
Appetite, n (%)		
Good (ref)	1	
Fair	1.26 (0.76, 2.08)	0.363
Bad	0.60 (0.19, 1.92)	0.386
Body Mass Index (BMI, kg/m^2^)		
Normal (ref)	1	
Underweight	7.68 (0.91, 64.97)	0.062
Overweight	0.39 (0.20, 0.76)	0.006
Obese	0.27 (0.15, 0.47)	< 0.001
**Health behaviors and Lifestyle characteristics**		
Smoking		
Non-smoker (ref)	1	
Ex-smoker	0.64 (0.34, 1.22)	0.175
Current smoker	0.68 (0.30, 1.53)	0.355
Level of physical activity (IPAQ short form)		
Low (ref)	1	
Moderate	0.69 (0.32, 1.50)	0.346
High	0.36 (0.16, 0.86)	0.020
**Nutritional status**		
Normal (ref)	1	
At risk or malnourished	2.25 (1.19, 4.29)	0.013
R Square	0.320	

*OR* odds ratio, *CI* confidence interval, *ref* Reference group of the categorical variable.

## Discussion

This secondary data analysis showed that the proportion of our cohort of Chinese community-dwelling older adults living alone who were at risk of sarcopenia and probable sarcopenia was 57.7% and 30%, respectively. Sarcopenia risk was positively associated with increasing age, sedentary lifestyle, risk of malnutrition, and being malnourished, while negatively associated with educational attainment. Additionally, overweight and obese individuals were less likely to develop sarcopenia, compared with underweight individuals and those with a normal weight.

We reported that over 50% of our cohort of community-dwelling older adults living alone was at risk of sarcopenia. This proportion is much higher than that reported in a cohort study conducted in Hong Kong using the SARC-F (3.8%) and the AWGS 2014 criteria (7.3%), a survey conducted in China using the AWGS 2014 criteria (6.4–19.3%), a study conducted in Singapore using dual-energy X-ray absorptiometry according to the AWGS 2019 criteria (13.6%), and a large prospective epidemiological study (United Kingdom Biobank) using a bioimpedance analysis according to the EWGSOP2 criteria (6.4%)^9,11,12,26,27^. This difference in sarcopenia prevalence could be partially attributed to the different diagnostic tools used in the studies. The 5-item SARC-F questionnaire is criticized by the low sensitivity (3.8‒4.8% in men and 8.2‒9.9% in women), thereby precluding its ability to detect individuals who actually have the condition^11^. A growing body of validation studies has demonstrated that the SARC-CalF has a better diagnostic performance with improved sensitivity (60.7–83.3%) and similar specificity (79–94.7%) in community-dwelling older adults, compared to the use of the SARC-F^14^. Incorporating the SARC-F, CC, and SARC-CalF is recommended to yield a more accurate identification of people at risk for of sarcopenia. Another possible contributor to this discrepancy could be the different ethnic groups and characteristics of the study populations. The studies conducted in western China, Singapore, and the United Kingdom recruited a considerable number of young-to-middle-aged participants, so their results may be susceptible to healthy responder bias, leading to a lower estimation of the sarcopenia prevalence^9,12,26^. Our findings demonstrated that sarcopenia could be common among older adults living alone thereby suggesting an urgent need to scale-up preventive and therapeutic interventions to reduce the burden of sarcopenia in this high-risk population.

The present study is among the few attempts to investigate the prevalence of probable sarcopenia according to the AWGS 2019 criteria. The prevalence of probable sarcopenia in the current study is consistent with that reported in a study conducted in Thailand (28.2%, N = 330) and Japan (30.3%, N = 552), but much higher than that reported in a study from Singapore (14.0%, N = 542) and in the UK Biobank study (5.3%, N = 499,046)^12,15,26,28^. Our findings suggest the usefulness of dynamometers for the easy detection of probable sarcopenia. Early detection of sarcopenia can contribute to timely referral for confirmatory diagnosis and implementation of primary preventive measures for the high-risk population.

Malnutrition, indicated by the MNA score, was found to be strongly associated with the risk of sarcopenia in the current study. The relationship between malnutrition and sarcopenia among older adults living alone is not well established, and the literature contains conflicting results on the association of the nutritional status with sarcopenia in older adults. For example, one study conducted in a geriatric outpatient population argued that the nutritional status was not associated with sarcopenia as defined according to the AWGS 2019 criteria (OR 2.16; 95% CI 0.93, 4.95; *p* = 0.07)^15^. However, Beaudart noted that malnutrition, defined according to the new European Society of Clinical Nutrition and Metabolism criteria, was significantly related to sarcopenia defined according to the EWGSOP2 criteria^29^. A recent prospective cohort study showed that malnourished individuals were at three-to-four-fold higher risk of developing sarcopenia compared to well-nourished individuals, during a 4-year follow-up^29^.

Malnutrition may contribute to the development of sarcopenia through complex pathways. In Chinese collectivist culture, eating is recognised as a social activity used to connect with friends and family. Older adults living alone are more likely to feel lonely, isolated, and depressed. These negative emotions probably reduce their appetite and dietary intake^6^. Poor appetite, measured by the Simplified Nutritional Appetite Questionnaire, was correlated with skeletal muscle mass index, which is recognised as an important measure of muscle quantity^30^. It was observed that 369 (94.6%) and 316 (81.0%) participants had meat and milk intakes lower than the daily recommendations of local dietary guidelines, respectively. Inadequate protein intake can limit muscle protein synthesis and increase muscle loss, thus accelerating the development of sarcopenia. Older adults who live alone tend to have fewer social relationships involving eating and are more likely to skip meals. Single-person households also face challenges in shopping for food and preparing meals, particularly in the case of individuals with sensory impairment and/or limited mobility. Hence, these individuals are less likely to prepare full, balanced meals. In our sample, 28.2% of the participants were considered malnourished or at risk of malnutrition, slightly higher than the 23.2% described for the Thai community-dwelling outpatient elderly and lower than that of the Turkish community-dwelling older population (32.3%)^31,32^. Malnutrition can also activate the immune system and increase the synthesis of pro-inflammatory cytokines, which in turn stimulates muscle catabolism, leading to body dysfunction and an increased risk of sarcopenia^33^. Regular assessment and monitoring of the nutritional status of older adults living alone should therefore be prioritised as part of routine health check-ups to identify individuals at risk of sarcopenia. Integrated interventions for older adults, including advocating the intake of a high-protein diet and cultivation of social circles and addressing their psychological needs, are potentially promising approaches to optimise their nutritional status and reduce their odds of sarcopenia.

The negative association between sarcopenia and a high level of physical activity indicates that physical activity has protective effects against sarcopenia for older adults living alone. The findings in the current study concur with previous research. A longitudinal study showed that increased moderate-to-vigorous physical activity was associated with 36% protection against sarcopenia incidence after 5 years^34^. A recent meta-analysis of nine studies with 2732 participants supported the protective role of physical activity against sarcopenia development (OR 0.45, 95% CI 0.37–0.55)^35^. It is, however, worth noting that a considerable proportion of older population living alone who reported a moderate level of physical activity still had sarcopenia (prevalence rate of 63.5%) and probable sarcopenia (56.4%). This result is in line with a previous study that exclusively targeted older adults with type 2 diabetes and reported that, compared with participants engaging in a high level of physical activities, those engaging in low and moderate levels of physical activities were likely to have a higher risk of sarcopenia (low: OR 2.15; 95% CI 1.07–4.35, *p* = 0.032; moderate: OR 2.23, 95% CI 1.07–4.66, *p* = 0.033)^13^. Moderate-intensity exercises improve muscle strength and functionality, but their effects in terms of counteracting age-related muscle loss may be limited^36^. These findings suggest that a high level of physical activity seems necessary to improve muscle mass and reverse sarcopenia development. As older adults living alone lack support and encouragement from an in-home caregiver, they are more vulnerable to the effects of insufficient rehabilitation. The appropriate intensity, dose, and type of rehabilitation required for older adults living alone should be comprehensively assessed.

Different approaches have been proposed for muscle mass adjustment, with the optimal estimation algorithm remaining unclear^1^. BMI calculated with respect to muscle mass performs better in predicting muscle strength, physical performance, daily activities, and frailty compared with BMI calculated with respect to height squared or total body mass^37^. In our study, compared with the participants with a normal weight, overweight and obese participants had a lower risk of sarcopenia development (*p* = 0.006 for overweight and *p* < 0.001 for obese), whereas underweight participants had a higher risk compared with those having a normal weight, with this association trending towards statistical significance (*p* = 0.062). This finding is line with previous findings that suggested an obesity paradox and showed that higher BMIs are associated with better outcomes among patients with a wide variety of chronic conditions, including sarcopenia. However, opposite direction of association between BMI with functional status was found in Turkish community-dwelling male older adults^38^. The association between excess weight and sarcopenia risk is still being debated. Increasing evidence corroborates that sarcopenic obesity is associated with accelerated functional decline, mortality, and increased risks of comorbidities, falls, disability, and long-term care placement^3^. The mechanism underlying the association between elevated body weight and sarcopenia risk warrants further investigation.

Multiple chronic diseases and polypharmacy significantly affect older individuals living alone, with CCI scores higher than those in the previous study^15^. In this study, multimorbidity was not associated with sarcopenia risk, which aligns with prior research conducted in Asia^13,15^. In contrast, a prospective analysis of 493,737 UK Biobank middle-aged and older adults demonstrated that multimorbidity was significantly associated with frailty, a condition that is interrelated with sarcopenia^26^. A recent multi-national population-based study that included 34,129 community-dwelling individuals aged over 50 years demonstrated that increased physical multimorbidity was associated with higher odds for weak handgrip strength. Handgrip strength is a clinically validated marker of sarcopenia risk^16^. Polypharmacy was also found to be associated with increased risk of sarcopenia among community-living elderly residents with type 2 diabetes in primary care clinics^13^. Future longitudinal studies recruiting older adults living alone from diverse settings are warranted to assess the relationship between multimorbidity and sarcopenia risk. These results have important implications for risk stratification and delivery of client-centred interventions to address the increasing challenges faced by older populations living alone.

Age and educational level were also found to be associated with the odds of sarcopenia in our study. This is in line with the findings of previous studies that suggested that older age is the most important risk factor for sarcopenia. Advancing age has been reported to be significantly associated with an increasing risk of developing sarcopenia (OR 2.28–6.87 for the 70–79 age group and 10.15–13.71 for the ≥ 80 age group)^15^. The age-related pathogenesis of sarcopenia is complex and may involve interactions of genetic predisposition with changes in hormone, inflammatory markers, and myostatin expression. In our study, the participants who received secondary school or higher education were less likely to develop sarcopenia. Similar findings were reported in other surveys^9,12^. Individuals with higher educational attainment may be more capable of recognising personal needs and locating and utilising relevant health information, which may in turn reduce their risk of sarcopenia. Overall, our findings suggest that special attention should be paid to individuals with advancing age and lower educational attainment to mitigate their risk of sarcopenia.

Several limitations of this study deserve to be mentioned. First, this was a cross-sectional study, a design that did not allow the derivation of any causal inference between the risk of sarcopenia and associated factors; thus, reverse causality between the risk and associated factors cannot be excluded. Longitudinal studies are necessary to disentangle the directionality of the relationship observed in the current study. Furthermore, the questionnaires used in this study captured physical activity engagement (7 days) and nutritional status (3 months) asynchronously. However, physical activity and nutritional status may change over time, potentially affecting the accuracy of our results. Given that appetite was measured using a simple 3-point Likert scale, rather than validated questionnaires, the negative association identified between appetite and sarcopenia risk should be interpreted with caution. Additionally, residual confounding effects (e.g., functionality) are still possible and future endeavours should consider potentially relevant conditions. Gene polymorphisms have been reported to be associated with muscular strength in older men living independently^39^. However, this is beyond the scope of this article and warrants future investigation to specifically focus on the genetic contribution to sarcopenia and declining physical fitness. Finally, older adults with higher BMIs tended to have better outcomes. The optimal lower threshold of BMI is higher for older individuals than their younger counterparts^38^. Moreover, BMI cut-off values vary between ethnic groups. In this study, BMI cut-off values for the Chinese population were used, potentially limiting the generalisability of this study.

## Conclusion

This study revealed that the proportion of Chinese community-dwelling older adults who live alone who were at risk of sarcopenia and those having probable sarcopenia is alarmingly high. This investigation is expected to be of great significance from a public health perspective. Age, educational level, and BMI were found to be factors associated with sarcopenia risk among older adults living alone. Maintaining a desirable nutritional status and engaging in a high level of physical activity may serve to protect or reduce the risk of developing sarcopenia. Our findings emphasise the urgent need to initiate aggressive screening and holistic lifestyle therapeutic intervention strategies for older adults living alone to mitigate the burden of sarcopenia.

## Data Availability

Data associated with this study can be obtained by reasonable request to the corresponding author (WKWS).
