# Author Correction: Sarcopenia risk and associated factors among Chinese community-dwelling older adults living alone

**DOI:** 10.1038/s41598-022-23029-8

**Published:** 2022-11-11

**Authors:** Li Cheng, Janet W. H. Sit, Helen Y. L. Chan, Kai Chow Choi, Regina K. Y. Cheung, Martin M. H. Wong, Francis Y. K. Li, Tin Yan Lee, Elina S. M. Fung, Keen Man Tai, Winnie K. W. So

**Affiliations:** 1 The School of Nursing, Sun Yat-Sen University, Guangzhou, China; 2 The Nethersole School of Nursing, Faculty of Medicine, The Chinese University of Hong Kong, Shatin, N.T., Hong Kong, Special Administrative Region China; 3 The Neighbourhood Advice-Action Council, North Point, Hong Kong, Special Administrative Region China

Correction to: *Scientific Reports* https://doi.org/10.1038/s41598-021-01614-7, published online 15 November 2021

The original version of this Article contained errors. *P* values in Tables 2 and 3 were dislocated.

The original Tables 2 and 3 with the accompanying legends appear below.

**Table 2:** Factors associated with risk of sarcopenia estimated by univariate ordinal logistic regression analysis (N = 390).

	Sarcopenia risk	
	OR (95% CI)	*P* value
**Socio-demographic characteristics**		
Age (per 10 years)	1.89 (1.42, 2.50)	< 0.001
Sex		
Male (ref)	1	
Female	0.95 (0.64, 1.40)	0.788
Marital status		
Single/divorced/separated/ widowed (ref)	1	
Married/cohabited	0.85 (0.53, 1.37)	0.508
Educational level		
No formal education (ref)	1	
Primary school	0.61 (0.38, 0.98)	0.040
Secondary school or above	0.43 (0.25, 0.71)	0.001
Employment status		
Retired (ref)	1	
Unemployed	1.33 (0.41, 4.30)	0.633
Have part-time/full-time job	1.70 (0.30, 9.51)	0.549
Monthly household income (HK$)		
< 6000 (ref)	1	
≥ 6000	3.05 (0.83, 11.16)	0.092
Received CSSA		
No (ref)	1	
Yes	1.26 (0.84, 1.89)	0.267
**Health status**		
Charlson comorbidity index (CCI)	1.15 (0.99, 1.33)	0.071
History of hypertension		
No (ref)	1	
Yes	0.14 (0.02, 0.16)	0.068
History of diabetes		
No (ref)	1	
Yes	1.10 (0.72, 1.69)	0.668
Visual impairment		
No (ref)	1	
A little	1.05 (0.67, 1.63)	0.843
A lot	2.43 (1.43, 4.13)	0.001
Hearing impairment		
Clear (ref)	1	
Fair	1.30 (0.82, 2.06)	0.264
Unclear	2.55 (1.32, 4.91)	0.005
Use of denture		
No (ref)	1	
Yes	1.04 (0.69, 1.57)	0.846
Difficulty in chewing food		
No (ref)	1	
Yes	1.55 (1.03, 2.33)	0.036
Appetite		
Good (ref)	1	
Fair	1.63 (1.08, 2.47)	0.020
Bad	1.15 (0.45, 2.94)	0.764
BMI		
Normal (ref)	1	
Underweight	5.22 (1.17, 23.41)	0.031
Overweight	0.38 (0.21, 0.67)	0.001
Obese	0.30 (0.19, 0.49)	< 0.001
**Health behaviors and lifestyle characteristics**		
Smoking status		
Non-smoker (ref)	1	
Ex-smoker	0.64 (0.38, 1.09)	0.097
Current smoker	1.04 (0.55, 1.96)	0.896
Drinking status		
Non-drinker (ref)	1	
Ex-drinker	1.04 (0.57, 1.93)	0.889
Current drinker	0.78 (0.41, 1.49)	0.463
Level of physical activity		
Low (ref)	1	
Moderate	0.79 (0.41, 1.49)	0.463
High	0.49 (0.24, 0.97)	0.040
Dietary intake: below recommendation of the dietary guidelines		
Grains (3 to 5 bowls per day)	0.82 (0.54, 1.23)	0.337
Vegetables (at least 3 servings per day)	0.91 (0.53, 1.55)	0.730
Fruits (at least 2 servings per day)	0.85 (0.55, 1.32)	0.479
Meats (5 to 6 taels per day)	0.30 (0.69, 1.63)	0.401
Milk (1 to 2 glass per day)	1.16 (0.70, 1.92)	0.570
**Nutritional status**		
Normal (ref)	1	
At risk or malnourished	3.82 (2.29, 6.35)	< 0.001
**Depressive symptoms**	0.73 (0.43, 1.23)	0.251

**Table 3:** Factors associated with risk of sarcopenia estimated by multivariable ordinal logistic regression analysis (N = 390).

	Odds ratio (95% CI)	*p*-value
**Socio-demographic characteristics**		
Age per 10 years	1.68 (1.17, 2.41)	0.005
Educational level		
No formal education (ref)	1	
Primary school	0.65 (0.36, 1.18)	0.160
Secondary school or above	0.50 (0.26, 0.96)	0.038
Monthly household income (HK$)		
< 6000 (ref)		
≥ 6000	1.07 (0.25, 4.65)	0.930
Charlson comorbidity index (CCI)	1.18 (0.99, 1.42)	0.070
History of hypertension		
No (ref)	1	
Yes	0.69 (0.41, 1.15)	0.153
Visual impairment		
No (ref)	1	
A little	0.71 (0.42, 1.21)	0.210
A lot	1.88 (0.94, 3.75)	0.072
Hearing impairment		
Clear (ref)	1	
Fair	0.94 (0.52, 1.67)	0.820
Unclear	1.26 (0.53, 2.99)	0.604
Difficulty in chewing food		
No (ref)	1	
Yes	1.48 (0.90, 2.42)	0.118
Appetite		
Good (ref)	1	
Fair	1.26 (0.76, 2.08)	0.363
Bad	0.60 (0.19, 1.92)	0.386
BMI		
Normal (ref)	1	
Underweight	7.68 (0.91, 64.97)	0.062
Overweight	0.39 (0.20, 0.76)	0.006
Obese	0.27 (0.15, 0.47)	< 0.001
**Health behaviors and Lifestyle characteristics**		
Smoking		
Non-smoker (ref)	1	
Ex-smoker	0.64 (0.34, 1.22)	0.175
Current smoker	0.68 (0.30, 1.53)	0.355
Level of physical activity (IPAQ short form)		
Low (ref)	1	
Moderate	0.69 (0.32, 1.50)	0.346
High	0.36 (0.16, 0.86)	0.020
**Nutritional status**		
Normal (ref)	1	
At risk or malnourished	2.25 (1.19, 4.29)	0.013
R Square	0.320	

The original Article has been corrected.

